# Neuro-Fuzzy Dynamic Position Prediction for Autonomous Work-Class ROV Docking

**DOI:** 10.3390/s20030693

**Published:** 2020-01-27

**Authors:** Petar Trslić, Edin Omerdic, Gerard Dooly, Daniel Toal

**Affiliations:** Centre for Robotics & Intelligent Systems, University of Limerick, V94 T9PX Limerick, Ireland; edin.omerdic@ul.ie (E.O.); gerard.dooly@ul.ie (G.D.); daniel.toal@ul.ie (D.T.)

**Keywords:** ANFIS, ROV docking, Position prediction

## Abstract

This paper presents a docking station heave motion prediction method for dynamic remotely operated vehicle (ROV) docking, based on the Adaptive Neuro-Fuzzy Inference System (ANFIS). Due to the limited power onboard the subsea vehicle, high hydrodynamic drag forces, and inertia, work-class ROVs are often unable to match the heave motion of a docking station suspended from a surface vessel. Therefore, the docking relies entirely on the experience of the ROV pilot to estimate heave motion, and on human-in-the-loop ROV control. However, such an approach is not available for autonomous docking. To address this problem, an ANFIS-based method for prediction of a docking station heave motion is proposed and presented. The performance of the network was evaluated on real-world reference trajectories recorded during offshore trials in the North Atlantic Ocean during January 2019. The hardware used during the trials included a work-class ROV with a cage type TMS, deployed using an A-frame launch and recovery system.

## 1. Introduction

In recent years, operations undertaken by unmanned underwater vehicles (UUVs) in the offshore energy sector are changing rapidly. This is driven by both offshore oil & gas (O&G) and the offshore wind sector where production platforms are pushed further off the coast, into areas of higher energy potential. However, considering significant expenditures related to the cost of the surface support vessel and crew, and with the production platforms in remote locations, the cost related to inspection, maintenance, and repair (IMR) tasks inevitably rise. Rising costs have resulted in the development and use of permanently deployed resident vehicle systems. Although the concept of permanently deployed vehicles exists in the literature for many years [1], only recently have we seen the introduction of commercial resident vehicles [2], with Oceaneering and IKM being industry leaders. Oceaneering developed E-ROV [3], a battery-powered, self-contained, work-class remotely operated vehicle (ROV), whereas IKM developed a fully electric R-ROV based on electric work-class ROV Merlin [4]. In general, such systems include a permanently deployed docking station which serves as a charging point, download/upload data link, and as mechanical protection for the resident vehicle [5].

However, within the O&G, and especially the offshore wind production field, multiple assets can be spread across more than 100 km${}^{2}$, which need to be continuously inspected for condition monitoring purposes. This has been partially addressed through the development of resident autonomous underwater vehicles (AUV) [6,7]. However, due to the limited intervention capabilities of resident AUV systems, many energy-intensive applications still require ROVs [8]. These restrictions are recognized, and use of collaborative platforms consisting of an autonomous surface vehicle (ASV) and ROV are seen as a potential solution [9,10]. Although commercially available solutions based on observation class ROVs exist [11], significant commercial uptake of the technology is not yet recorded.

Resident ROVs operating from shore fundamentally demand a high bandwidth, low latency communications link that is often unavailable, thus high levels of automation are needed. This is especially important for time-critical tasks since manual operation from the shore due to the mentioned communication problems is not viable. One of the essential time-critical tasks in resident vehicle operation is the docking of the vehicle at the end of the mission. Autonomous docking of UUVs is a well-researched area, with main focus on docking to a static docking station, both for ROV docking to a tether management system (TMS) [12,13] and AUV docking to a docking station [14,15]. However, a TMS suspended from a surface platform such as a surface vessel, presents a highly dynamic system, with wave height and period dictating the viability of launch and recovery operations [16].

Although docking of UUVs to a moving docking station is reported, the research is mainly focused on an AUV docking and on compensation of disturbances in the horizontal plane (e.g., cross-current), while assuming minimal docking station heave oscillations. Recovering of an AUV by another AUV in shallow water is presented in [17]. The system consists of a “mother” AUV with a funnel shaped docking station attached to its body, designed to accommodate launch and recovery of the “daughter” AUV. A docking to an active docking station is presented in [18]. The paper presents a cooperative guidance system for the AUV docking, whereas the system consists of a funnel shaped receptacle with an active heading adjustment. A USV-based automated launch and recovery system (LARS) for AUVs is presented in [19]. The recovery system is based on the deployment of a thin line with a depressor wing from the surface vessel, whereas the AUV is equipped with a pincer-type mechanism for latching. Another fixed-wing depressor-based solution is presented in [20], where a funnel shaped DS is attached to the depressor and towed by a surface vehicle at a constant speed, whereas the AUV intercepts the docking station and performs the docking. The ROV launch and recovery from an ASV has been previously reported in [21]. The system included Video Ray micro-ROV launch and recovery directly, without the docking station.

One of the major limitations of the autonomous ROV docking to a suspended TMS is the TMS heave motion, which can exceed amplitudes of 3 m. Those limits were recognized and reported during previous trials, which included, a first autonomous docking of a work-class ROV to a suspended TMS [22]. Findings acquired during those trials investigating TMS behavior in a real-world environment and associated docking limitations, have served as a motivation for this paper. Although a certain amount of misalignment between the ROV and the TMS is allowed during docking, work-class ROVs are generally underpowered, and not agile enough to match the TMS heave motion. During the manual docking process this is compensated by the pilot’s TMS heave motion analysis, prediction of TMS heave motion, and experience. However, considering the offshore marine renewable energy (MRE) sector with devices placed in areas of strong wind, current, and tides, while acknowledging the previously mentioned communication related problems, manual docking operation from shore is not viable. Also the autonomous docking of a ROV to a garage supported beneath a floating platform is expected to be very challenging. Therefore, to allow for autonomous work-class ROV docking in higher sea states a TMS heave motion prediction method has been developed. To the author’s knowledge, this is the first time TMS heave position prediction has been proposed.

This research paper presents development and evaluation of the method for suspended TMS heave motion prediction, based on an adaptive neuro-fuzzy inference system (ANFIS). In addition, the paper discusses the mapping of surface vehicle motions to the suspended docking station coordinate frame. The prediction of TMS heave motion has the potential benefits of allowing autonomous docking in higher sea states, extending the ROV operational weather windows, and reducing the misalignment between the ROV and TMS during the docking process, thus reducing the impact on the ROV system and extending the ROV operational life. Furthermore, the method has a dual benefit of being applicable to autonomous docking or as an aiding tool for the pilot. The ANFIS performance is evaluated on a real-world dataset recorded using a work-class ROV with corresponding cage type TMS, deployed during offshore trials in the North Atlantic Ocean.

## 2. The Hardware

**Figure 1:**
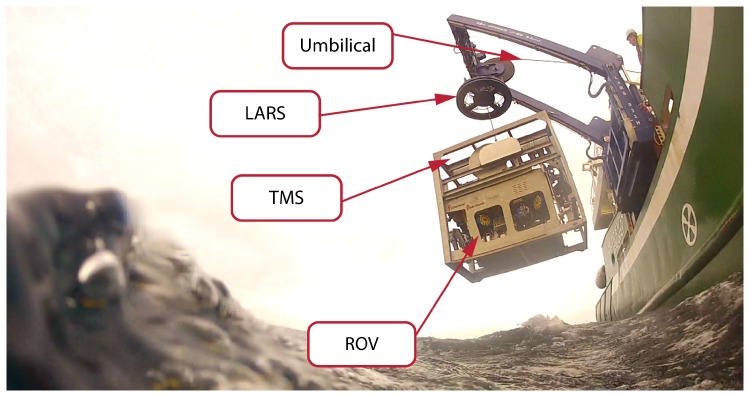
The system deployment during offshore trials in the North Atlantic Ocean in January 2019. The system consists of Launch and Recovery System (LARS), Tether management System (TMS), and work-class ROV Comanche.

The significant restricting factor in ROV operations and associated operational weather windows relates to the launch and recovery of the vehicle. This includes both launch and recovery of the ROV from the vessel to the sea, and launch and recovery of the ROV from the TMS while underwater. In this paper problems associated with the latter are discussed. The main systems involved in the ROV deployment trials are a surface vessel, a launch and recovery system (LARS), a tether management system (TMS) and the ROV itself. The overview of the system used during the trials is shown in Figure 1, whereas the basic technical specification is given in Table 1. The ROV used during the trials is a work class Comanche ROV developed by Sub-Atlantic and Forum Energy Technologies, which is one of the standard ROVs used in the offshore sector. The ROV is equipped with two Schilling Orion hydraulic arms and is capable of operating in depth up to 2000 m. The vehicle weights approximately 1.6 tons and can achieve a maximum speed of 2.5 knots.

There are various definitions of a tether management system, and although the TMS is in essence only a subsea tether-handling mechanism, by common convention and according to The ROV Manual [23] it is “typically described as the entire subsea mechanism from the end of the umbilical (umbilical termination to the clump/depressor weight, cage, or top hat) to the beginning of the soft tether”. There are two main types of tether management systems, the top hat TMS and the cage type TMS. Although most discussed problems related to TMS heave compensation could relate to both types of TMS, in the remainder of this paper when using term TMS, a cage-type TMS is assumed.

There are three main functions of the tether management system: (1) to manage, usually neutrally buoyant soft tether, which connects the TMS and the ROV, and provide power and communications to the ROV; (2) to protect the vehicle against damage during ROV deployment and recovery phase; and (3) to act as a clump weight to absorb the cross-section drag that would be otherwise introduced to a tether connecting ship and the ROV. Therefore, the ROV is completely relieved of the tether drag from the surface to the working depth, which is important due to the limited power on-board the ROV available for vehicle drag compensation caused by sea currents. To act as a clump weight, the TMS has to be negatively buoyant; thus, as a consequence, the ship’s motion is directly translated to the TMS through a steel reinforced umbilical connecting the TMS with the ship. The TMS used during the trials is a cage type, side entry TMS and weights approximately 2.2 tons.

The LARS system is used as the overboarding equipment, and its primary role is to move ROV from the deck and deploy it safely. The most typically used LARS is the A-frame type, such as the one used during the trials. The system weights around 12 tons and contains 2.2 km of umbilical. On one side the LARS is connected to the control cabin and ship’s power supply, and on the other side to the TMS through the umbilical. The umbilical used for TMS deployment is steel reinforced for lifting to/from the water, and it provides the power and communication link between the ROV and the control cabin. Vessels that are specially designed for ROV operations may include the moonpool or Cage & Rail LARS system [9] for deployment. However, such equipment is more complicated, thus more expensive, and generally is used as a permanent feature on the vessel. The control cabin is considered the ROV control center with multiple PCs dedicated to ROV control, sonar imaging, image acquisition, processing, etc. Therefore, the data acquired by ROV sensors is sent to the control cabin on the surface where all computation related to the ROV operations is conducted. During the trials the complete system was deployed on the 66 m long research vessel RV Celtic Explorer.

### 2.1. The TMS Motion Analysis

The TMS with the ROV is usually deployed from a surface vessel or floating platform which is exposed to various disturbances such as waves, currents, wind, tides, and others. As the surface vessel for TMS deployment is the main source of the TMS motion, it is necessary to understand all the disturbances introduced to the surface vessel and how they map to the TMS. As the disturbances act on all six degrees of freedom (DOF) of the surface vessel, and considering the TMS is connected with the vessel through the non-elastic umbilical, those motions couple to the TMS directly. Therefore, the primary goal during the TMS deployment is to minimize the impact of those disturbances on a surface vessel.

**Figure 2:**
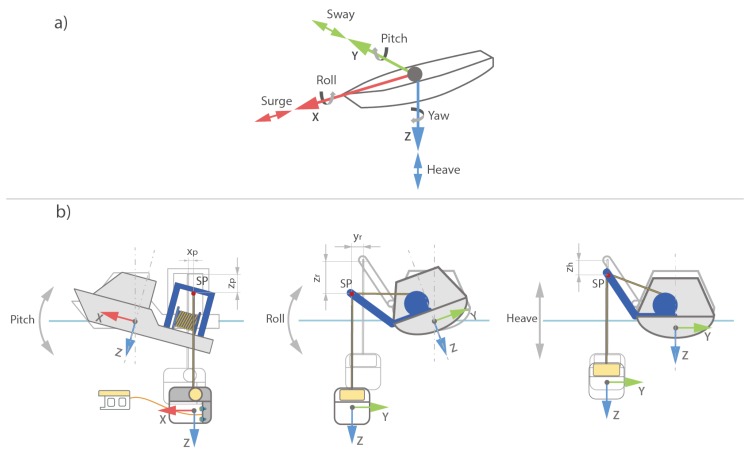
Ship motion mapped to the TMS heave motion. (**a**) Vessel’s six degrees of freedom; (**b**) Deployment vessel pitch and roll mapped in TMS coordinate frame.

Figure 2a shows a vessel’s six degrees of freedom. Work-class ROV operations generally imply use of a deployment vessel with dynamic positioning (DP) capabilities. A DP vessel is capable of holding position and heading, thus sea current and wind-related disturbances are bounded by vessel’s surge, sway, and yaw control. However, the sea-wave height and period have a direct impact on vessel’s roll, pitch, and heave, which cannot be directly compensated for; thus, these remaining three DOF translate to TMS principally as a heave motion. Figure 2b shows the ship motion translated to the TMS.

Given the ship heave, roll, and pitch, a total TMS heave displacement $z_{\mathrm{TMS}}$ is calculated as

(1) $$z_{\mathrm{TMS}}=z_{h}+z_{r}+z_{p}$$

where $z_{h}$ is heave of the TMS directly proportional to the heave of the ship, $z_{r}$ is heave of TMS due to the ship roll motion, and $z_{p}$ is the heave of the TMS due to the ship pitch. As shown in the figure, to reduce the TMS heave, the suspension point SP should ideally be placed close to the ship pitch and roll axis. The vessels designed specifically for ROV operations exploit this with integrated, moonpool LARS or ship door LARS [16,23] systems. However, the most typical LARS is the A-frame type, as shown in Figure 1.

The docking station is relatively stable on the roll and pitch axis since the TMS center of gravity is below the point where TMS is attached to the umbilical, thus positive longitudinal and lateral stability is achieved. Sea current generally rotates the TMS around the yaw axis, until TMS reaches the orientation that creates the least amount of drag. However, TMS yaw is easily controllable with two or more thrusters attached to the TMS. There are two sources of TMS surge and sway: (1) surface vessel surge and sway which depends on DP capability, and (2) the TMS surge and sway caused by displacement of suspension point $y_{r}$ and $x_{p}$ due to vessel’s roll and pitch, which, for relatively small angles can be neglected. In addition, the TMS inertia, length of the deployed umbilical, and water act together as a damper, thus they reduce surge and sway oscillations.

In summary, the suspended TMS heave displacement $Z_{TMS}$ depends on the surface vessel motion, which depends on various parameters, such as vessel’s size and type, the weather conditions, the location of the LARS on vessel’s deck, the size of the LARS, etc. Although it is not possible to measure all the variables, as explained in the next section, the ROV pilot is able to perform the docking maneuver successfully based solely on the visual estimation and prediction of TMS heave displacement. In practice, during manual docking, the ROV pilot estimates the displacement by observing the video feed either from the ROV or the TMS camera to estimate relative motion between the two. A similar approach is presented in this paper, with an ANFIS-based TMS heave displacement prediction $Z_{TMS}$ up to *t* seconds in the future, based on previous $Z_{TMS}$ measurements. There are various ways to measure TMS heave displacement, such as using depth sensor, altimeter, acoustic positioning system, vision system, etc. As the dataset acquired during the trials consists of TMS depth measurements, and the ROV uses depth control, ANFIS is trained to predict TMS depth. In the next section a manual ROV docking is presented, and importance of TMS heave prediction for the docking is discussed.

### 2.2. ROV Docking

Docking of a ROV system is one of the most critical tasks dictated by operation weather windows. It introduces a high risk of ROV damage, and it can be a highly stressful operation for ROV pilot in challenging sea conditions. The docking maneuver can be divided into three stages: (1) the preparation stage, (2) the ROV approach and TMS heave estimation stage, and (3) contact stage. Manual ROV docking into a cage type TMS starts with the ROV stern facing the entrance of the TMS.

**Figure 3:**
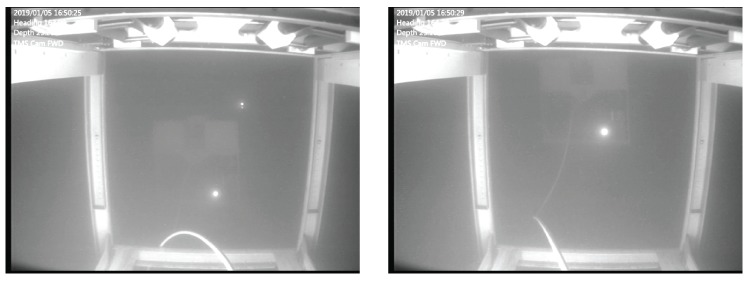
The TMS heaving while ROV holds constant depth. Photo taken during the trials.

In the first stage, the ROV heading, depth and the lateral position should be aligned with the TMS. In general, the TMS mechanically allows for certain vertical and horizontal misalignment due to the funnel-shaped entrance. Therefore, for relatively small TMS heave amplitudes, approximately $heave_{max}\leq 1$ m peak-to-peak for the system presented in the paper, the ROV is able to dock while holding mean TMS depth. Figure 3 shows the TMS heaving prior to a manual docking maneuver, whereas the ROV holds constant depth.

**Figure 4:**
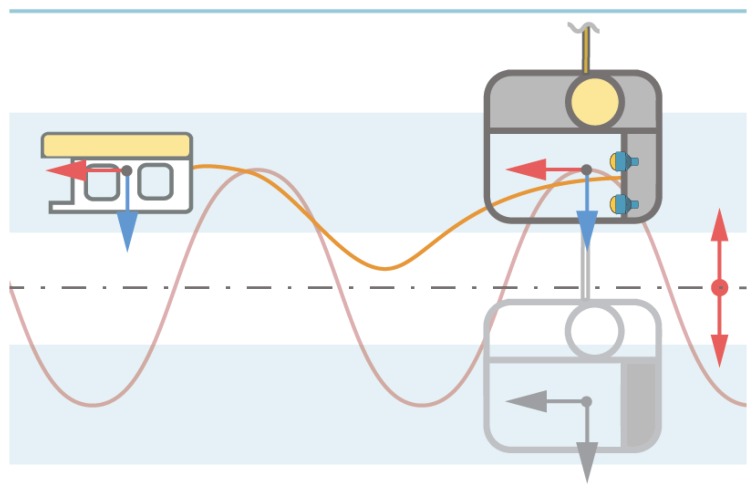
The ROV docking procedure. Red line presents the TMS heave motion, whereas blue shaded area shows optimal docking position with minimal TMS heave speed.

However, $heave_{max}$ is often exceeded, thus in the second stage after the alignment, the vehicle approaches the TMS entrance slowly, while the ROV pilot estimates TMS heave amplitude and frequency. Generally, the work-class ROVs are not agile enough to match the TMS heave motion due to the weight, and high drag forces associated with the ROV’s large cross-sectional area. To overcome the problem, the pilot positions the ROV to the docking depth that covers either top or the bottom half of the TMS heave range, as shown in Figure 4. As the TMS reaches the minimum or the maximum heave value, it slows down, until it entirely stops and reverses direction. The pilot exploits this knowledge and positions the ROV at a corresponding depth, as the shaded area in Figure 4 shows.

Although the TMS heave amplitude and frequency are not fixed, once the ROV is in the approximately correct area, the ROV depth can be fine adjusted quickly. To allow for large ROV inertia, the docking maneuver is typically started before the TMS reaches the optimal position for docking. Therefore, the pilot must predict the TMS position based on experience, and current and previous observations, and undertake a decision in a fraction of second while controlling the ROV.

The third stage includes the contact between the ROV and the TMS, and finishes with the ROV docked. As, in general, it is not possible to compensate for all the motion and align ROV perfectly with the TMS, the docking still includes rough or bumpy contact; however, to a much reduced extent.

## 3. Adaptive Neuro-Fuzzy Inference System - ANFIS

This section describes the implementation of the adaptive neuro-fuzzy inference system (ANFIS) for TMS motion prediction. ANFIS is an adaptive neural network which is equivalent to a fuzzy inference system (FIS) first time introduced in [24]. With ANFIS, a set of fuzzy if-then rules is identified, with membership function parameters tuned through a hybrid learning algorithm.

**Figure 5:**
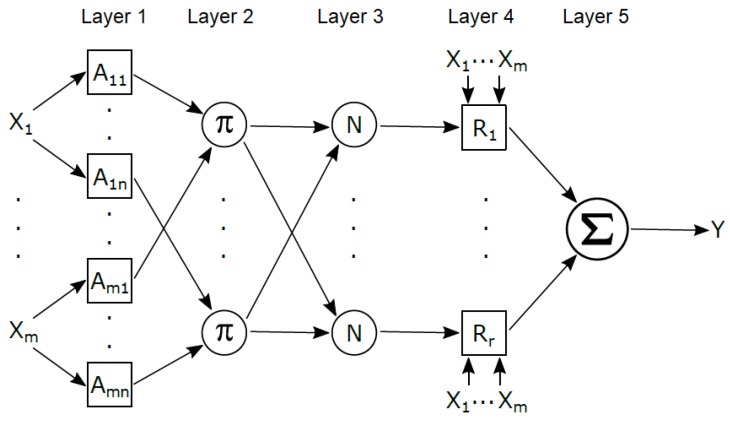
ANFIS network framework architecture.

Figure 5 shows the ANFIS network architecture that consists of five layers. Each of *m* inputs (X) is assigned with *n* fuzzy membership functions described with linguistic labels (A), constituting *r* rules (R). Each node in the first layer is adaptive and specifies the degree to which a given input satisfies the fuzzy membership function related with that node. The first layer is called the “fuzzification” layer, while parameters in this layer are called *premise parameters*. In the second layer a firing strength for each rule is determined. Every node in this layer is fixed and labeled π, while the node performs multiplication of the incoming signals. Every node in the third layer is fixed and normalizes the firing strengths of the previous layer. The fourth layer is called the “defuzzification” layer. This layer consists of adaptive nodes and it involves computing the weighted consequent value for each given rule. Parameters in this layer are called *consequent parameters*. The node in the last layer performs summation of all incoming signals.

Various authors reported use of ANFIS for modeling nonlinear functions such as motion prediction of moving targets [25,26,27], predicting stock market return [28], electricity price forecasting [29], and various other. In addition, ANFIS performs exceptionally well when predicting chaotic time series. This is demonstrated in [24], where comparison between ANFIS, cascaded-correlation neural network, backpropagation MLP, autoregressive model, and other networks have been given. As the position prediction of the TMS belongs to the same class of problems, the use of ANFIS should be considered and evaluated.

The tuning of the network is done using an existing dataset consisting of input–output pairs, while the network tries to model the function which relates input to output. By using past values of the heave displacement $z{}_{TMS}$ up to time *t*, ANFIS is used to predict the future value of the $z{}_{TMS}\left(t+P\right)$. As $z{}_{TMS}$ is measured using a depth sensor, this is achieved by mapping a dataset of known TMS depth values using *D* points of the time series spaced Δ apart as

(2) $$\left[z_{TMS}\left(t-\left(D-1\right)\Delta \right),\ldots ,z{}_{TMS}\left(t-\Delta \right),z{}_{TMS}\left(t\right)\right],$$

to a predicted value in future $z{}_{TMS}\left(t+P\right)$. Therefore, for parameters D=3, Δ=1.5, P=2, one input–output ANFIS pair is given by

(3) $$\left[z{}_{TMS}\left(t-3\right),z{}_{TMS}\left(t-1.5\right),z{}_{TMS}\left(t\right)\right],\left[z{}_{TMS}\left(t+2\right)\right]$$

where $\left[z{}_{TMS}\left(t-3\right),z{}_{TMS}\left(t-1.5\right),z{}_{TMS}\left(t\right)\right]$ is the input which consists of the last D=3 depth measurements, spaced Δ=1.5 s apart, mapped to the output $\left[z{}_{TMS}\left(t+2\right)\right]$, which presents the predicted TMS depth value P=2 s in the future.

The ANFIS training and evaluation has been done on a prerecorded dataset. The data used for ANFIS training and evaluation was recorded during the offshore trials that took place in the North Atlantic Ocean during January 2019. The TMS depth was recorded using a depth sensor attached to the TMS frame. The sensor used during the trials was UV-SVP by Valeport. It is a conventional commercial unit that offers pressure, sound velocity and temperature measurements in one housing. Technical specification of the unit is given in Table 2.

For the given task, different process values are involved in constructing an efficient and reliable ANFIS network. This variables include size of the input–output dataset pairs (training dataset length), number of membership functions per input MF, the number of training epochs NE, number of training points *D*, how far in future TMS position is to be predicted *P*, spacing between the points Δ, sensor sampling frequency $f{}_{s}$, etc.

Although there are guidelines about the ANFIS training process [24,30], as with other neural networks, there are still no specific rules to estimate the optimal parameters for the network training. The parameters can vary greatly and depend on the quality of data and complexity of the problem, thus it relies on trial and error experiments. If such an approach is not possible, various techniques for estimating optimal ANFIS tuning parameters have been presented before [31,32]. Although extensive trial and error experiments have been performed to investigate the effect of various network parameters, the focus of the paper is as follows.

- Evaluate ANFIS performance for TMS position prediction.
- Analyze the network training time, and consider real-time ANFIS training.
- Investigate the influence of the depth sensor sample rate on ANFIS performance.

Therefore, in the next section, an overview of the best network configuration is given at the start, followed by an ANFIS overall performance evaluation.

## 4. Results

The scope of the paper is to investigate and evaluate the usage of an ANFIS network for the prediction of TMS heave motion. Prior to the evaluation, an optimal network configuration and training parameters should be determined. The optimal ANFIS for the given problem achieves minimum error RMSE with a minimum network training duration. The RMSE is a root mean square error between predicted future TMS depth value and measured value at that time, and it is considered as one of network performance measures. As explained in Section 2, the TMS heave motion depends on the LARS type and the deployment vessel type. Once the network is trained, the performance is reduced if certain LARS, TMS, and deployment vessel combination are changed. As the goal is to enable the possibility to retrofit the solution to the existing ROV fleet, network training on-site is necessary. In addition, constantly changing sea conditions should be considered. Although the trained network can perform well for a certain amount of time after training, a change in sea conditions influences the network performance, thus the online ANFIS training is considered and tested.

### 4.1. Optimal ANFIS Configuration for TMS Heave Prediction

To find optimal ANFIS training parameters the experiments included varying the following parameters.

- Dataset length in range 50 to 600 s.
- Number of membership functions MF per input in range 2 to 5.
- Number of previous measurements *D* in range 1 to 12.
- Spacing between previous measurements Δ in range 0.5 to 5 s.
- Number of training epochs NE in range 1 to 250.
- Prediction time *P* in range 0.5 to 5 s.

Although extensive experiments have been conducted to determine the best set of parameters, only main experiments related to the ANFIS parameters estimation have been presented, considering the main scope of the paper.

The effect of each parameter on ANFIS performance and training duration is shown in Table 3. In general, the input selection criteria is based on [33], which is based on the assumption that the ANFIS network with the smallest RMSE after one epoch of training has a better potential to achieve a lower RMSE given more training epochs.

The relationship between the number of training points *D* and the number of epochs, RMSE, and duration of the training process, is shown in Table 4. The data in the table is divided in two major columns by the number of epochs used for training. The left column provides results after only one epoch of training, whereas the right column shows RMS errors and training duration at the epoch with minimum $RMSE_{CHK}$. As shown in the table, both the training $RMSE_{TRN}$ and the checking $RMSE_{CHK}$ error decreased until D=4. For D=5, the training error keeps decreasing, while the checking error grows. In addition, the difference between the two grows significantly at D=5, which is the sign of network overfitting and must be avoided. Although in general the network performed better after more than 1 epoch, the difference in $RMSE_{CHK}$ is not significant. For example, at D=4, after one epoch the checking error is $RMSE_{CHK}=0.0422624$ m, and the minimum error is achieved at epoch 63 with $RMSE_{CHK}=0.0421802$ m. The difference between the two is negligible, whereas the duration of the training extended ten times from 0.053 s to 0.53 s. The number of membership functions is MF=2 as the increase in MF leads to exponential growth of fuzzy rules, thus the training time grows exponentially. In addition, no reduction in $RMSE_{CHK}$ has been achieved.

From extensive experiments, the optimal ANFIS input parameters for the given task are found as D=4, Δ=1, NE=1, MF=2 per input. Therefore, the best network performance is achieved by using the last four consecutive measurements (D=4), spaced one second apart (Δ=1), using only one training epoch, with two membership functions per input MF=2. The same network configuration performed best for various values of prediction time P.

**Figure 6:**
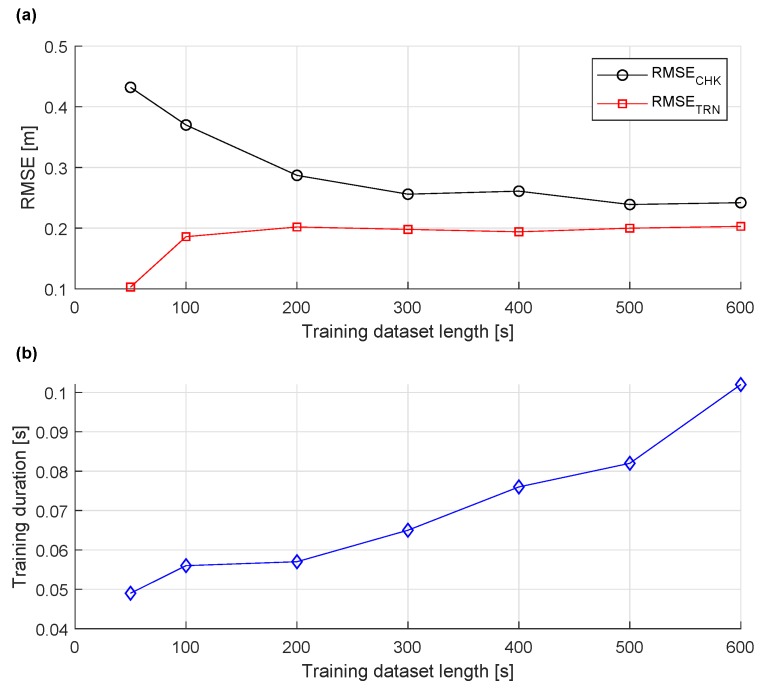
The relationship between length of training dataset and checking error $RMSE_{CHK}$, and training error $RMSE_{TRN}$ (**a**) and training duration (**b**).

The amount of data used for online training should be taken into consideration as well since a larger training dataset increases the ANFIS training duration. The effect of training dataset length on ANFIS training cycle duration, training $RMSE_{TRN}$, and checking $RMSE_{CHK}$ error is shown in Figure 6. Multiple experiments have been conducted with the training dataset increased from 50 to 600 s. As shown in Figure 6a, for 50 s of the training data, $RMSE_{TRN}$ is relatively low while $RMSE_{CHK}$ is high, which is a sign of network overfitting the training data, and should be avoided. The overfitting is caused by small amount of training data points compared to number of ANFIS modifiable parameters. Up to 200 s of training dataset, most of the $RMSE_{CHK}$ is reduced. After that point, training duration grows with little improvement in $RMSE_{CHK}$ as shown in Figure 6b.

### 4.2. The TMS Heave Prediction Based on ANFIS

After network parameters (D,Δ,EN,MF, dataset length) were evaluated as in Section 4.1, multiple tests have been performed to evaluate the ANFIS performance for the TMS heave prediction. Prior to the ANFIS performance test, it is necessary to establish the evaluation criteria. Although hard contact between the ROV and the TMS is expected during the docking in a harsh environment, the main objective is to reduce rough contact to the minimum. This is achieved by reducing the misalignment between the ROV and the TMS at the moment of contact during docking. In general, the TMS and the ROV are designed in such a way to allow a certain amount of misalignment for easier docking. However, this should be minimized to reduce the risk of ROV damage, which leads to increased operational expenditure (OPEX) costs. The amount of allowed misalignment is determined experimentally. During the previously reported offshore trials [22], the work-class ROV was autonomously docked multiple times with the TMS peak to peak heave amplitude of 1.1 m, while the ROV operated at mean TMS depth. Therefore, the particular ROV-TMS configuration shown in Figure 1, and Figure 3 tolerates vertical misalignment of ±0.55 m. Thus, the ANFIS is considered as performing well when the difference between predicted and measured value is:

(4) $$err_{TMS}=\left|z_{TMS}p\left(t+P\right)-z_{TMS}m\left(t+P\right)\right|\leq 0.55\;m,$$

where $z_{TMS}p\left(t+P\right)$ is the predicted TMS depth, and $z_{TMS}m\left(t+P\right)$ is the measured TMS depth at the same time. However, sometimes during manual docking the TMS heaves more than the pilot predicts, thus the misalignment between the TMS and the ROV is larger than $err_{TMS}>0.55$ m, and the maneuver has to be aborted. Therefore, as an additional ANFIS performance indicator a mean absolute error (MAE) is calculated as

(5) $$MAE=\frac{\sum_{i+1}^{N}a_{i}}{N},$$

for $a_{i}=1$, if $err_{TMS_{i}}\leq 0.55$ m and $a_{i}=0$ for $err_{TMS_{i}}>0.55$ m, which essentially shows the percentage of time the error between predicted and measured TMS, depth was $err_{TMS}\leq 0.55$ m. Due to equipment involved in offshore operations being particularly expensive, the network is considered performing well when MAE≥95%.

**Figure 7:**
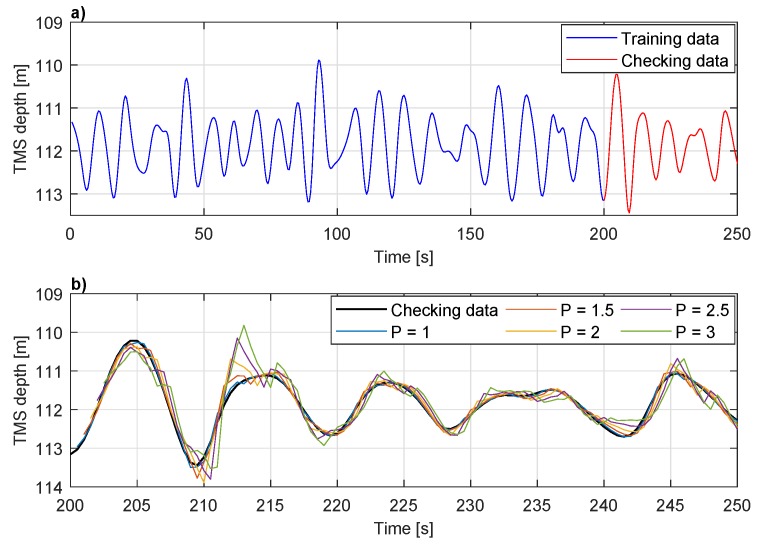
Dataset used for ANFIS evaluation. (**a**) First 200 s of data is used for a training, while 50 s of data is used for checking. (**b**) Performance of ANFIS predicting TMS depth between 1 s and 3 s in future compared to checking data.

The depth measurements of the cage type TMS suspended from the ship RV Celtic Explorer during the trials in the North Atlantic Ocean are shown in Figure 7a. The TMS depth recorded over 150 s period, ranges between 110 and 113 m with the mean depth of approximately 112 m. The depth sensor sampling frequency was $f_{s}=2$ Hz. When working with neural networks, it is common to use a fraction of recorded data for the training, whereas the remaining fraction of the data is used for the network validation. The ANFIS training stage included the first 200 s of the data, while the next 50 s of data is used for the checking stage. The experimentally determined optimal ANFIS parameters are D=4, Δ=1 s, NE=1, and MF=2 per input. Figure 7b, above, shows only checking data of the same dataset (last 50 s) compared to the predicted TMS depth values. The TMS position prediction 1 s in future $z{}_{TMS}\left(t+1\right)$ (continuous blue line) has the smallest deviation from the measured value, while the difference between checking data and data predicted 3 s ahead is significant.

**Figure 8:**
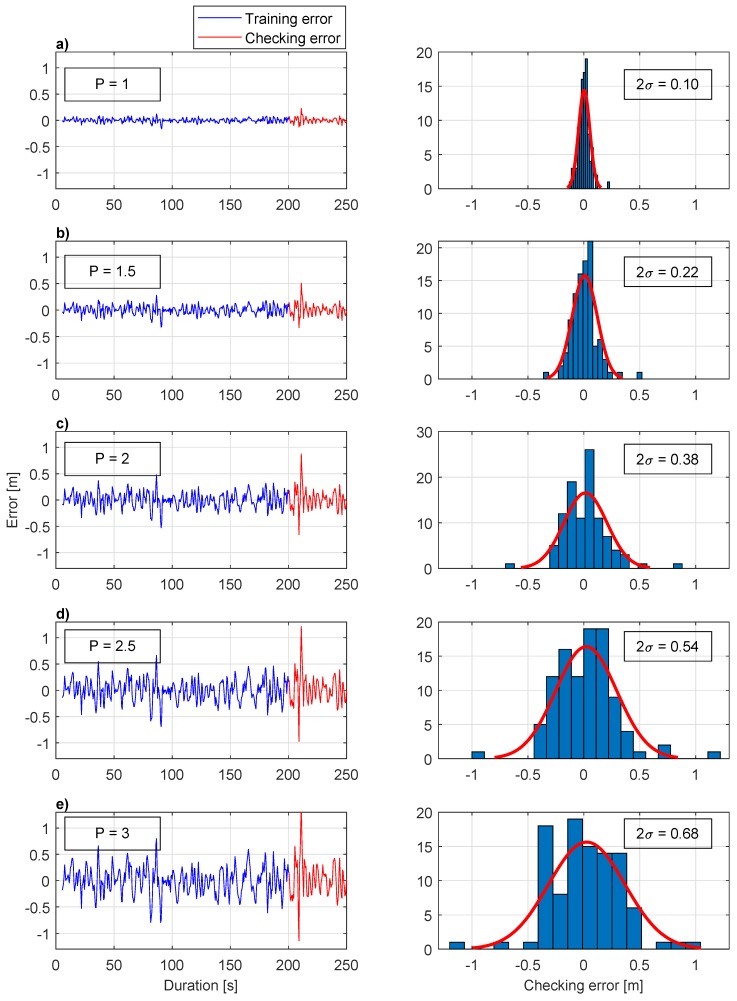
The error between predicted and real TMS depth with corresponding checking error distributions. (**a**) The TMS depth prediction 1 s in future; (**b**) The TMS depth prediction 1.5 s in future; (**c**) The TMS depth prediction 2 s in future; (**d**) The TMS depth prediction 2.5 s in future; (**e**) The TMS depth prediction 3 s in future.

Table 5 and Figure 8 show the ANFIS performance for TMS depth prediction up to P=3 s in future. ANFIS performs exceptionally well for predicting $z{}_{TMS}\left(t+1\right)$, with two standard deviations of the error only 2σ=0.10 m. For P=1.5 s, 2σ reached the value of 0.23 m, and it continues to grow until it reaches the value 2σ=0.55 m for P=2.5 s, with MAE=95.05%. By increasing prediction time further to P = 3 s, the error grows further, and the criteria MAE≥95%, is not satisfied. Therefore, the results of the experiment showed that ANFIS could be successfully used for the TMS heave position prediction $z_{TMS}$ up to 2.5 s in future for the particular TMS − ROV setup of the experiments while keeping prediction error below 0.55 m in 95% of the time.

### 4.3. Online ANFIS Training

**Figure 9:**
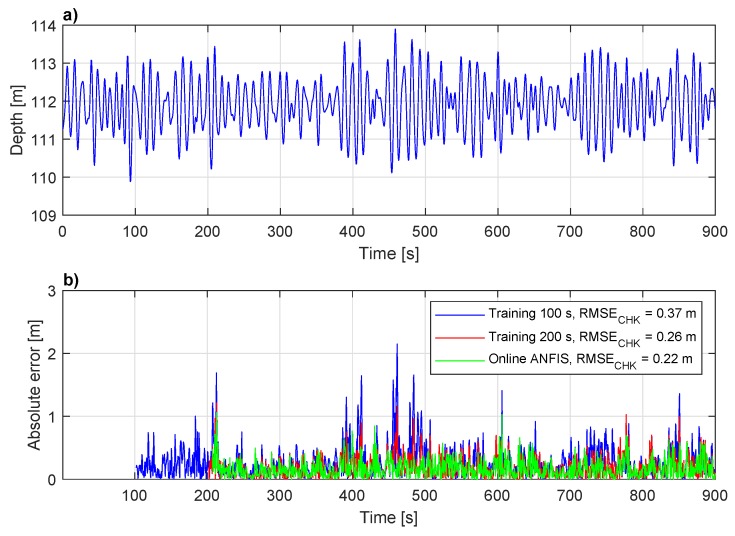
Online trained ANFIS performance. (**a**) The TMS depth dataset used for ANFIS training and evaluation; (**b**) Comparison between ANFIS trained on first 100 s of data (blue), trained on first 200 s of data (red), and trained online using last 200 s of data before each prediction step (green).

The performance of the ANFIS network is further investigated. As mentioned previously, the sea conditions continuously change, thus the performance of the network trained on one set of the data degrades with changes in TMS heave frequency and/or amplitude. Figure 9a shows the TMS depth recorded over a 900 s period using the depth sensor sampling frequency $f_{s}=2$ Hz. Three ANFIS networks, composing of the same structure (D=4, MF=2, Δ=1), are trained to predict the TMS depth 2.5 s in future (P=2.5).

The first ANFIS network is trained using only the first 100 s of the data, which means that the prediction model of the TMS behavior has been built based only on those measurements. Similar to other neural networks, once a new input data is out of the range the neural network has been trained for, big errors occur. Therefore, as shown in Figure 9b, at time 200 s, between 380 and 420 s, and between 450 and 500 s there is a significant error (blue line). For comparison, the second ANFIS network (red line) has been trained using the first 200 s of the data, therefore it is more “experienced”, and has been able to predict the TMS behavior better than the first one. However, a sudden change in TMS depth amplitude between 380 and 420 s, and between 450 and 500 s, still caused high prediction errors. To compensate for this, the ANFIS network should be trained considering the latest available data. With the online ANFIS (green line), the prediction model of the TMS behavior has been recalculated and updated after each TMS depth measurement, using the latest 200 s of the data. For example, at time 380 s the online ANFIS gives approximately the same error as the second ANFIS, however, at time 420 s the prediction error has been significantly reduced. Therefore, at time 450 s based on the last 200 s of “experience”, which also includes the depth measurements between 380 and 420 s, the ANFIS already “expects” a sudden change in the TMS depth, thus between 450 and 500 s the prediction error is reduced. In summary, with the online ANFIS training approach the network is trained continuously, while taking into account the latest acquired data from the TMS depth sensor. As the figure shows, the online ANFIS training further improved TMS depth prediction. While overall $RMSE_{CHK}$ is reduced (only 0.04 m), the error spikes are significantly reduced.

### 4.4. Depth Sensor Sample Rate

In the previous subsection, the optimum training dataset duration is experimentally identified to be 200 s. However, the amount of data points recorded during the 200 s time period depends on the depth sensor sampling frequency $f_{s}$. Ideally, the sampling frequency of the sensor should be high enough to accurately capture relevant frequency specter, but not too high to cause long ANFIS training time.

**Figure 10:**
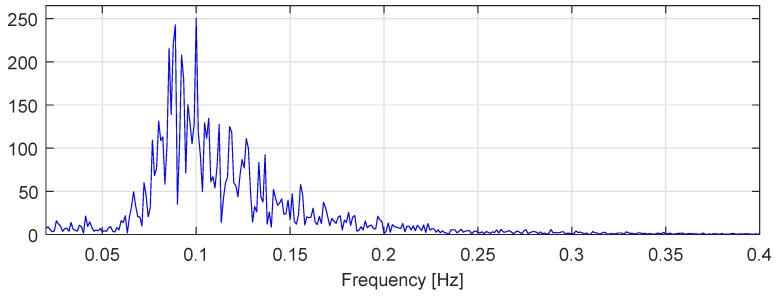
The TMS heave frequency spectre.

Figure 10 shows a frequency specter of the dataset previously illustrated in Figure 9a. The frequency range of the TMS heave motion $f_{TMS}$ is between 0.05 and 0.25 Hz, with the most prominent frequencies $f_{TMS}$ is tetween 0.08 and 0.1 Hz. Therefore, the minimum sensor sampling frequency to cover full TMS heave frequency specter, is by Shannon–Nyquist theorem $f_{s}min=f_{TMS}max*2=0.5\;\mathrm{Hz}$. This was further inspected. Table 6. shows the relation between ANFIS performance and different sensor sampling frequencies. In each case, the network was trained with 200 s of data and evaluated on the remaining fraction using the same parameters as follows, D=4, Δ=1, NE=1, MF=2, and P=2.

With the sensor sampling frequency lower than $f_{s}min$, the actual TMS depth change is not recorded accurately. In addition, the low number of datapoints leads to overfitting, with $RMSE_{CHK}$ over 2 m at $f_{s}=0.25$ Hz. With the increase of the sampling frequency to $f_{s}=f_{s}min=0.5$ Hz, the number of datapoints doubled, and overfitting is avoided. By doubling $f_{s}$ to 1 Hz, $RMSE_{CHK}$ is further reduced to 0.25 m, which is a big improvement over the previous case. The sensor sampling frequency $f_{s}=2$ Hz provided best results with $RMSE_{CHK}=0.179$ m, whereas the ANFIS training time cost is only 0.057 s, and compared to the prediction time of 2.5 s this is negligible. Further increase in sensor sampling frequency leads to an increase in the ANFIS training duration, while the contribution in prediction performance is minimal.

## 5. Discussion and Conclusions

This paper presents a suspended TMS depth prediction method for ROV docking, based on the Adaptive Neuro-Fuzzy Inference System (ANFIS). The method is used to extend the ROV operational weather windows, reduce operational expenses, and reduce ROV damage due to the harsh docking. The docking of underpowered work-class ROVs to a heaving TMS relies entirely on the ROV pilot experience in estimating TMS heave motion, which is not available for autonomous and resident underwater vehicles. With large ROV inertia and drag forces acting against it, the ROV is not agile enough to match TMS heave motion, thus the docking Figure starts before the ROV and the TMS align. The method presents an addition to the suite of technologies required for dynamic autonomous work-class ROV docking and is beyond the current state of the art in work-class ROV technology. In addition, the method has the potential for retrofitting to the existing ROV fleet, to be used as a ROV pilot aiding tool, and it does not require additional hardware.

The method has been tested in the field on real-world data recorded during the offshore trials in North Atlantic Ocean, during January 2019. The trials during research cruise CE-19001 [34] included work-class ROV Étaín, with the corresponding TMS deployed from the research vessel RV Celtic Explorer, using A-frame LARS. The trained ANFIS network showed excellent performance when predicting the TMS depth up to 2.5 s into the future with RMSE=0.22 m, and with 97% of errors below maximum allowed vertical misalignment between the ROV and the TMS $err_{TMS_{i}}\leq 0.55$ m. Further modification of the TMS entrance with a funnel-shaped receptacle would allow for larger misalignment, thus, extending the operational docking window.

The future work includes detailed time response ROV analysis, modeling the ROV docking strategy decision process, and implementation with the ROV and OceanRings [35,36] suite of smart technologies developed in house at the Centre for Robotics and Intelligent Systems, to extend the ROV autonomous docking capability. 

## Figures and Tables

**Table 1:** Technical specification of the system.

	Description	Dimensions L × W × H (m)	Weight (t)
**Control Cabin**	Reinforced container used as ROV control centre	6 × 2.4 × 2.4	6.5
**LARS**	A - frame type, 2200 m steel enforced umbilical, ϕ 25.4 mm	5.5 × 2.8 × 3.2	12
**TMS**	Cage-type, 400 m soft tether, ϕ 21 mm	2.9 × 1.8 × 2.5	2.2
**ROV**	Middle size ROV capable of inspection, maintenance and repair tasks	2.1 × 1.3 × 1.25	1.6
**Ship**	Research Vessel	Length - 66 m	Displacement - 2425 t

**Table 2:** The Valeport UV-SVP sensor technical specifications.

	Pressure (bar)	Temperature (${}^{\circ }$C)	Sound Velocity (m/s)
**Operating range**	300	−5 to +35	1375 to 1900
**System resolution**	0.001% of range	0.001	0.001
**System accuracy**	± 0.01% of range	± 0.01	± 0.02

**Table 3:** Relationship between different parameters, ANFIS training duration, and performance.

Parameter		Training Duration	${\mathrm{RMSE}}_{\mathrm{CHK}}$	${\mathrm{RMSE}}_{\mathrm{TRN}}$	Generates Overfitting
Dataset length	↑	↑	↓	↓	No
Number of MF	↑	↑	↓	↓	Yes
Number of D	↑	↑	↓	↓	Yes
Prediction time P	↑	-	↑	-	No
Number of Epochs	↑	↑	↓	↓	Yes

**Table 4:** The relationship between training points D, number of training epochs, RMSE, and duration of the training process.

D	Epoch 1			Epoch with Minimal Check Error			
	$RMSE_{TRN}$ (m)	$RMSE_{CHK}$ (m)	Duration (s)	Epoch	$RMSE_{TRN}$ (m)	$RMSE_{TRN}$ (m)	Duration (s)
2	0.103221	0.0864964	0.03305	1	0.103221	0.0864964	0.03305
3	0.0613137	0.0577967	0.050134	189	0.0569494	0.0560394	0.370561
**4**	**0.0374812**	**0.0422624**	**0.052966**	63	0.0337162	0.0421802	0.526817
5	0.0296526	0.0973706	0.102934	42	0.0276815	0.0861169	1.863842

**Table 5:** ANFIS performance for predicting TMS depth up to P=3 s in future.

P (s)	MAE (%)	${\mathrm{RMSE}}_{\mathrm{CHK}}$ (m)	${\mathrm{RMSE}}_{\mathrm{TRN}}$ (m)	2σ (m)
1	100	0.050	0.038	0.101
1.5	99.01	0.112	0.083	0.225
2	97.03	0.192	0.142	0.384
2.5	95.05	0.273	0.202	0.546
3	93.07	0.340	0.250	0.680

**Table 6:** The relationship between depth sensor sampling frequency and ANFIS performance.

Sampling Frequency $f_{s}$ (Hz)	Number of Datapoints in 200 s	Training Duration (s)	${\mathrm{RMSE}}_{\mathrm{TRN}}$ (m)	${\mathrm{RMSE}}_{\mathrm{CHK}}$ (m)
0.25	50	0.032	0.007	2.397
0.5	100	0.035	0.152	0.543
1	200	0.041	0.179	0.280
2	400	0.060	0.142	0.176
4	800	0.079	0.141	0.179
8	1600	0.144	0.158	0.156
16	3200	0.322	0.158	0.157

## Abbreviations

The following abbreviations are used in this manuscript:

ANFIS	Adaptive neuro-fuzzy inference system
ASV	Autonomous surface vehicle
DOF	Degree of freedom
DP	Dynamic positioning
FIS	Fuzzy inference system
LARS	Launch and recovery system
MAE	Mean average error
MRE	Marine renewable energy
O&G	Oil and gas
OPEX	Operating expenses
RMSE	Root mean squared error
ROV	Remotely operated vehicle
TMS	Tether management system
UUV	Unmanned underwater vehicle
